# Comparing Glial Fibrillary Acidic Protein (GFAP) in Serum and Plasma Following Mild Traumatic Brain Injury in Older Adults

**DOI:** 10.3389/fneur.2020.01054

**Published:** 2020-09-18

**Authors:** Nathan A. Huebschmann, Teemu M. Luoto, Justin E. Karr, Ksenia Berghem, Kaj Blennow, Henrik Zetterberg, Nicholas J. Ashton, Joel Simrén, Jussi P. Posti, Jessica M. Gill, Grant L. Iverson

**Affiliations:** ^1^Department of Physical Medicine and Rehabilitation, Spaulding Rehabilitation Hospital, Charlestown, MA, United States; ^2^Sports Concussion Program, MassGeneral Hospital for Children, Boston, MA, United States; ^3^Department of Neurosurgery, Tampere University Hospital and Tampere University, Tampere, Finland; ^4^Department of Physical Medicine and Rehabilitation, Harvard Medical School, Boston, MA, United States; ^5^Home Base, A Red Sox Foundation and Massachusetts General Hospital Program, Charlestown, MA, United States; ^6^Spaulding Research Institute, Charlestown, MA, United States; ^7^Department of Radiology, Medical Imaging Centre, Tampere University Hospital, Tampere, Finland; ^8^Department of Psychiatry and Neurochemistry, Institute of Neuroscience and Physiology, The Sahlgrenska Academy, University of Gothenburg, Mölndal, Sweden; ^9^Clinical Neurochemistry Laboratory, Sahlgrenska University Hospital, Mölndal, Sweden; ^10^UK Dementia Research Institute, University College London, London, United Kingdom; ^11^Department of Neurodegenerative Disease, University College London Institute of Neurology, Queen Square, London, United Kingdom; ^12^Wallenberg Centre for Molecular and Translational Medicine, University of Gothenburg, Gothenburg, Sweden; ^13^Maurice Wohl Clinical Neuroscience Institute, Institute of Psychiatry, Psychology & Neuroscience, King's College London, London, United Kingdom; ^14^National Institute of Health Research Biomedical Research Centre for Mental Health & Biomedical Research Unit for Dementia, South London & Maudsley National Health Service Foundation, London, United Kingdom; ^15^Neurocenter, Department of Neurosurgery, Turku Brain Injury Center, Turku University Hospital, University of Turku, Turku, Finland; ^16^Division of Intramural Research, National Institutes of Health, Bethesda, MD, United States

**Keywords:** traumatic brain injuries, glial fibrillary acidic protein, plasma, serum, computed tomography

## Abstract

**Objective:** Identification and validation of blood-based biomarkers for the diagnosis and prognosis of mild traumatic brain injury (mTBI) is of critical importance. There have been calls for more research on mTBI in older adults. We compared blood-based protein marker glial fibrillary acidic protein (GFAP) concentrations in serum and in plasma within the same cohort of older adults and assessed their ability to discriminate between individuals based on intracranial abnormalities and functional outcome following mTBI.

**Methods:** A sample of 121 older adults [≥50 years old with head computed tomography (CT), *n* = 92] seeking medical care for a head injury [Glasgow Coma Scale scores of 14 (*n* = 6; 5.0%) or 15 (*n* = 115; 95.0%)] were enrolled from the emergency department (ED). The mean time between injury and blood sampling was 3.4 h (*SD* = 2.1; range = 0.5–11.7). Serum GFAP concentration was measured first using the Human Neurology 4-Plex Assay, while plasma GFAP concentration was later measured using the GFAP Discovery Kit, both on an HD-1 Single molecule array (Simoa) instrument. Glasgow Outcome Scale-Extended was assessed 1 week after injury.

**Results:** Both serum and plasma GFAP levels were significantly higher in those with abnormal CT scans compared to those with normal head CT scans (plasma: *U* = 1,198, *p* < 0.001; serum: *U* = 1,253, *p* < 0.001). The ability to discriminate those with and without intracranial abnormalities was comparable between serum (AUC = 0.814) and plasma (AUC = 0.778). In the total sample, GFAP concentrations were considerably higher in plasma than in serum (Wilcoxon signed-rank test *z* = 0.42, *p* < 0.001, *r* = 0.42). Serum and plasma GFAP levels were highly correlated in the total sample and within all subgroups (Spearman's *rho* range: 0.826–0.907). Both serum and plasma GFAP levels were significantly higher in those with poor compared to good functional outcome (serum: *U* = 1,625, *p* = 0.002; plasma: *U* = 1,539, *p* = 0.013). Neither plasma (AUC = 0.653) nor serum (AUC = 0.690) GFAP were adequate predictors of functional outcome 1 week after injury.

**Conclusions:** Despite differences in concentration, serum and plasma GFAP levels were highly correlated and had similar discriminability between those with and without intracranial abnormalities on head CT following an mTBI. Neither serum nor plasma GFAP had adequate discriminability to identify patients who would have poor functional outcome.

## Introduction

Glial fibrillary acidic protein (GFAP) concentration is increased following a traumatic brain injury (TBI), in studies of patients with predominantly mild to moderate injuries (1–6). There is some evidence that GFAP is associated with, and may be able to predict, unfavorable outcome following TBI (7–10). GFAP is a 50 k-Da intermediate filament protein that is highly abundant in the cytoskeleton of astrocytes (11, 12). Following plasma membrane damage secondary to neurotrauma, GFAP is released into the interstitial fluid, and enters the bloodstream by crossing the blood-brain barrier, which is compromised following TBI (13–15) or via the glymphatic system (14, 16). GFAP has been shown to be detectable within 1 h of injury (3, 17, 18), continues to rise and appears to peak within 20–24 h (3, 18), and then declines over 72 h (3), with a biological half-life of 24–48 h (19). Many studies have examined the utility of GFAP for identifying patients with intracranial abnormalities following TBI (20). GFAP is considered useful for this purpose given that it is specific to brain injury (7, 21–23) and has a relatively long half-life compared to other biomarkers (19). The Food and Drug Administration (FDA) recently approved GFAP and ubiquitin carboxyl-terminal hydrolase L1 (UCH-L1) for use in the emergency department (ED) to screen for traumatic intracranial abnormalities and aid clinical decisions regarding acute head CT scanning (24).

A large body of evidence from samples obtained from patients with mild to moderate TBIs suggests that GFAP in both serum (1–3, 18, 25, 26) and plasma (4–6, 27) can discriminate between those with and without acute traumatic abnormalities on head CT, outperforming both UCHL-1 (4) and S100B (2). Despite extensive research examining GFAP, a comprehensive, direct comparison of serum and plasma GFAP levels from the same patient sample is lacking. The purpose of the present study is to compare serum and plasma GFAP levels following mTBI within the same sample of subjects using two different and widely used assays. We chose to examine a convenience sample of older adults with mild TBIs (mTBI) because (i) there have been calls for more research focused on mTBI in older adults (28, 29), (ii) they have pre-existing neurological conditions that can influence biomarker results (30), and (iii) there is evidence that they have higher levels of GFAP following injury than younger adults, as well as generally have more prolonged recoveries (31, 32). We hypothesized that plasma and serum GFAP levels would (i) be highly correlated, (ii) have similar ability to discriminate between those with and without acute traumatic intracranial abnormalities on head CT, and (iii) have similar associations with functional outcome in older adults with a mTBI.

## Materials and Methods

### Participants

The data used for secondary analyses in the present study were part of a larger prospective study that aimed to clinically validate the Scandinavian Guidelines for Initial Management of Minimal, Mild, and Moderate Head Injuries in Adults in the Emergency Department (ED) of the Tampere University Hospital (33). Tampere University Hospital is the only neurosurgical referral hospital in the district, and the ED provides health services for approximately 470,000 residents from 22 municipalities, both urban and rural. All adult patients aged 18 or older, with an acute traumatic head injury, seen within 24 h of injury, were eligible for inclusion. The minimum criteria for TBI were as follows: either blunt injury to the head or acceleration/deceleration type injury resulting in witnessed loss of consciousness, disorientation, or amnesia and an initial Glasgow Coma Scale (GCS) score of 13–15. Over a 1-year period from November 2015 to November 2016, 325 patients provided written consent to be included in the study and 225 had both serum and plasma analyzed for GFAP. From these 225, we selected a sample of 121 older adults (≥50 years; 51.2% men) with a suspected mTBI, based on a Glasgow Coma Scale (GCS) of 14–15 upon presentation to the ED, who had blood drawn within 12 h of injury for the present study. The sample for the present study was limited to older adults in part because (i) nearly all patients who presented to the ED who had abnormal head CT scans in the cohort were, coincidentally, older adults; (ii) GFAP has been shown to increase with age (32, 34); and (iii) GFAP was recently approved to screen for intracranial abnormalities (24), making this a convenience study of older adults.

### Functional Outcome Assessment

Participants were administered the Finnish-language Glasgow Outcome Scale-Extended (GOS-E) (35) at 1 week post-injury by a trained research nurse. The 1-week time point was selected for the original S100B validation study to assess if the patients developed early complications that could have been avoided with initial head CT scanning in the ED. The GOS-E ranges from 1 to 8, with higher ratings corresponding to better functional outcome following injury during this subacute time period. The GOS-E was dichotomized with GOS-E of 7 and 8 considered *Good Outcome* and a GOS-E of 6 or lower considered *Poor Outcome*.

### Computed Tomography

Non-contrast head CT was performed with a 64-row CT scanner (GE, Lightspeed VCT, WI, USA). Clinical judgment was used to decide whether to perform head CT, but decisions mainly adhered to the Scandinavian Guidelines (36). We did not rely on the interpretation from the on-call radiologist at the time of injury. A neuroradiologist, for research purposes, reviewed and systematically coded all CT findings based on the Common Data Elements (37). To verify the reliability of the head CT findings, an independent neuroradiologist re-interpreted 10% of the CT-scanned patients from the original prospective cohort (33) with the same common data elements. The interrater intraclass correlation for a normal vs. abnormal head CT was 0.879 (95% CI = 0.719–0.948, *p* < 0.001), indicating excellent agreement. Pre-existing and acute traumatic lesions were coded. The following traumatic lesions were considered as intracranial abnormalities on head CT: skull fracture, epidural hematoma, extraaxial hematoma, subdural hematoma, traumatic subarachnoid hemorrhage, vascular dissection, traumatic aneurysm, venous sinus injury, midline shift, cisternal compression, fourth ventricle shift/effacement, contusion, intracerebral hemorrhage, intraventricular hemorrhage, diffuse axonal injury, traumatic axonal injury, penetrating injuries, craniocervical junction/brainstem injury, edema, brain swelling, ischemia/infarction/hypoxic-ischemic injury. Based on head CT, participants were divided into those who did not undergo head CT, those with intracranial abnormalities on head CT, and those without intracranial abnormalities on head CT.

### Blood Sampling and Analytics

Venous blood samples were collected within 12 h of injury. The blood samples were collected in Tampere between November 2015 and November 2016. Serum GFAP levels were measured first on March 12, 2018 in a research laboratory in Mölndal, Sweden using the Human Neurology 4-Plex Assay (Quanterix, Billerica, MA) on an HD-1 Single molecule array (Simoa) instrument according to instructions from the manufacturer (Quanterix, Billerica, MA). The lower limit of detection for GFAP was 0.221 pg/mL and the lower limit of quantification was 0.467 pg/mL. Calibrators were run in duplicates while samples were run in singlicates. Two quality control samples were run in duplicates in the beginning and the end of each run, showing a repeatability of 5.8% and intermediate precision of 5.8% at 79.8 pg/mL, and a repeatability of 4.9% and intermediate precision of 6.2% at 87.5 pg/mL.

Plasma GFAP levels were analyzed on September 14–15, 2019, again in Mölndal, Sweden using the GFAP Discovery Kit (Quanterix, Billerica, MA) on an HD-1 Simoa instrument according to instructions from the manufacturer (Quanterix, Billerica, MA). The lower limit of detection for GFAP was 0.211 pg/mL and the lower limit of quantification was 0.686 pg/mL. Calibrators were run in duplicates while samples were run in singlicates. Samples were run with a 4-fold dilution and results have been compensated for this dilution. Two internal quality control samples were run in duplicates in the beginning and end of each run. For a quality control sample with a concentration of 76.3 pg/mL, repeatability was 7.6% and intermediate precision was 11.3%, whereas for a quality control sample with a concentration of 204.2 pg/mL, repeatability was 6.8% and intermediate precision was 12.8%.

### Ethical Approval

Ethics approval was obtained from the Ethics Committee of Pirkanmaa Hospital District, Tampere, Finland (ethical code: R15045). Written informed consent was obtained from all included study participants after participants were provided with necessary information about the study in both oral and written form.

### Statistical Analyses

All analyses were conducted for the total sample (*N* = 121) and designated subgroups based on head CT findings and functional outcome. Non-parametric tests were used given that both serum and plasma GFAP levels were non-normally distributed. Within-person comparisons of serum and plasma GFAP levels were conducted using Wilcoxon signed-rank tests, with effect size *r* calculated for each analysis by dividing the *z*-statistic by the square root of the sample size (38). This effect size can be interpreted as small (*r* = 0.10), medium (*r* = 0.30), and large (*r* = 0.50) (39). Cohen's *d* has also been reported and can be interpreted as small (*d* = 0.20), medium (*d* = 0.50), and large (*d* = 0.80) (39), but this effect size assumes normality and may not accurately reflect the magnitude of difference between groups. Non-parametric correlations (i.e., Spearman's *rho*) were also calculated to examine the relationship between age and GFAP levels, time to blood sampling and GFAP levels, and GFAP levels across serum and plasma. Mann-Whitney *U* tests were conducted to compare participants based on head CT findings (i.e., positive vs. negative) and outcome (i.e., poor vs. good), with effect size *r* again calculated to quantify the magnitude of the effect. Receiver Operator Characteristic (ROC) curve analyses were conducted to determine the sensitivity and specificity of the serum and plasma GFAP levels at discriminating between participants with and without intracranial abnormalities on head CT and participants with good and poor outcome based on GOS-E. The Area Under the Curve (AUC) was calculated for each ROC analysis, under a non-parametric assumption, with an accompanying standard error (*SE*) and 95% confidence interval (CI). AUC values were interpreted as acceptable (AUC = 0.70–0.79), excellent (AUC = 0.80–0.89), and outstanding (AUC ≥ 0.90) at discriminating between groups (40). Statistical analyses were conducted using SPSS version 24 and the MedCalc Statistical Software version 19.17 (Bland-Altman Plot, ROC analyses, and Passing Bablock regression). Bland-Altman analyses were used to illustrate the agreement between the two quantitative measurements. Passing Bablock regression is a non-parametric method for estimating a linear regression line and testing whether the intercept is zero and the slope is one, which would illustrate that two measurement systems were yielding the same values.

## Results

### Patient Characteristics, Blood Sampling, and Imaging Findings

The total sample (*n* = 121; 51.2% men) had a mean age of 75.1 years old (*SD* = 11.9) and a median age of 76.0 with an interquartile range (*IQR*) of 68.0 to 84.5 (full age range = 50.0–100.0). All participants had GCS scores of 14 (*n* = 6; 5.0%) or 15 (*n* = 115; 95.0%) in the ED. The mean time to blood sampling was 3.4 h (*SD* = 2.1; *Md* = 2.9, *IQR* = 1.9–4.5, range = 0.5–11.7). The injury characteristics of the total sample and imaging findings are presented in Table 1. Intracranial abnormalities were identified in 18.2% (*n* = 22) of the total sample and 23.9% of those who underwent head CT (*n* = 92). The imaging findings for those who underwent head CT were as follows: skull fracture (*n* = 2; 2.2%), extra-axial hematoma (*n* = 18; 19.6%), acute subdural hematoma (*n* = 10; 10.9%), traumatic subarachnoid hemorrhage (*n* = 9; 9.8%), intraventricular hemorrhage (*n* = 1; 1.1%), midline shift (supratentorial) (*n* = 2; 2.2%), contusion (*n* = 4; 4.3%), and traumatic axonal injury (*n* = 3; 3.3%). No patient had an isolated skull fracture.

**Table 1:** Injury characteristics of the sample and imaging findings.

	**Yes**	**No**	**Unknown**
	***n*, %**	***n*, %**	***n*, %**
Loss of consciousness-witnessed/suspected	45, 37.2%	58, 47.9%	18, 14.9%
Post-traumatic seizure	0, 0%	105, 86.6%	16, 13.2%
Post-traumatic amnesia	41, 33.9%	71, 58.7%	9, 7.4%
Focal neurological deficit	11, 9.1%	108, 89.3%	2, 1.7%
Vomited 2 times or more	2, 1.7%	109, 90.1%	10, 8.3%
Headache	50, 41.3%	62, 51.2%	9, 7.4%
Alcohol intoxication at time of injury	39, 32.2%	75, 62.0%	7, 5.8%
Neurosurgery (craniotomy)	2, 1.7%	119, 98.3%	–
Other surgery	0, 0%	121, 100%	–
Acute traumatic lesion on head computed tomography	22, 18.2%	78, 64.5%	29, 24.0%

*No patient had an isolated skull fracture*.

Participants were divided into subgroups based on head CT findings, including positive head CT (*n* = 22; 40.9% men; *M* = 81.1 years old, *SD* = 9.4, *IQR* = 72.8–89.0, range = 61.0–96.0), negative head CT (*n* = 70; 47.1% men; *M* = 75.1 years old, *SD* = 12.2, *IQR* = 66.8–84.3, range = 50.0–100.0), and head CT not conducted (*n* = 29; 69.0% men; *M* = 70.4 years old, *SD* = 11.1, *IQR* = 61.0–79.0, range = 50.0–91.0). Participants were also divided into subgroups based on GOS-E into good outcome (*n* = 31; 58.1% men; *M* = 71.3 years old, *SD* = 11.0, *IQR* = 66.0–78.0, range = 50.0–91.0) and poor outcome (*n* = 76; 46.1% men; *M* = 76.5 years old, *SD* = 11.7, *IQR* = 70.3–85.8, range = 50.0–96.0). The distribution of GOS-E scores for the entire sample was as follows: 1: *n* = 0, 0%; 2: *n* = 1, 0.8%; 3: *n* = 17, 14.3%; 4: *n* = 28, 23.5%; 5: *n* = 2, 1.7%; 6: *n* = 6, 5.0%; 7: *n* = 22, 18.5%; 8: *n* = 31, 26.1%; and missing: *n* = 12, 10.1%. Two participants sustained repeat head injuries within 1 week of their initial presentation to the ED and were excluded from the 1-week functional outcome analyses.

### Findings in the Total Sample

Descriptive statistics for serum and plasma GFAP concentrations in the total sample are presented in Table 2. GFAP concentration values were considerably greater in plasma than in serum for the total sample (*z* = 0.42, *p* < 0.001, *r* = 0.42, medium to large effect size). Serum and plasma GFAP concentrations in the total sample were highly correlated, but the values were not redundant (*rho* = 0.886).

**Table 2:** Comparison of GFAP concentrations in serum and plasma in the total sample and head CT subgroups.

	**Total sample**		**No head CT**		**Normal head CT**		**Abnormal head CT**	
	**(*****N*** **= 121)**		**(*****n*** **= 29)**		**(*****n*** **= 70)**		**(*****n*** **= 22)**	
	**Plasma**	**Serum**	**Plasma**	**Serum**	**Plasma**	**Serum**	**Plasma**	**Serum**
**Descriptive statistics (pg/mL)**								
Mean	635.9	497.0	400.3	401.4	459.1	344.5	1509.2	1108.2
Median	326.8	272.1	207.3	217.1	315.9	263.0	928.6	787.8
Standard deviation	972.6	700.5	790.1	867.2	535.7	412.4	1627.8	872.3
Interquartile range	189.9–595.1	157.2–503.3	137.1–337.0	122.3–302.1	176.3–564.9	147.8–433.5	411.0–2035.6	353.5–2096.6
Range	52.2–6838.6	32.6–4813.8	52.2–4420.0	32.6–4813.8	63.4–4027.7	48.7–3382.4	139.6–6838.6	114.4–2640.7
**Difference score (plasma – serum)**								
Mean	138.9		−1.1		114.6		401.0	
Median	28.0		0.65		37.7		230.1	
Standard deviation	509.2		90.7		237.9		1088.0	
Interquartile range	−15.5 to 140.4		−21.6 to 39.6		−9.0 to 144.4		−54.7 to 612.0	
Range	−1612.0 to 4197.8		−393.8 to 129.4		−118.6 to 1328.7		−1612.0 to 4197.83	
Percentage with plasma > serum	64.5%		55.2%		68.6%		63.6%	
**Group comparisons**								
Wilcoxson signed ranks test (*z*)	4.62 (*p* < 0.001)		0.75 (*p* = 0.456)		4.46 (*p* < 0.001)		2.09 (*p* = 0.036)	
Effect sizes (*r*)	0.42		0.14		0.53		0.45	
Effect sizes (Cohen's *d*)	0.16		0.001		0.24		0.31	
**Spearman correlations**								
Age and GFAP level	0.495^**^	0.540^**^	0.489^**^	0.540^**^	0.508^**^	0.631^**^	−0.028	−0.061
Time to blood sampling	0.243^**^	0.206^*^	0.315	0.308	0.158	0.074	0.455^*^	0.387
Values from two assays	0.886^**^		0.907^**^		0.862^**^		0.826^**^	

* p < 0.05 and

** *p < 0.01; CT, computed tomography; GFAP, glial fibrillary acidic protein*.

**Figure 1:**
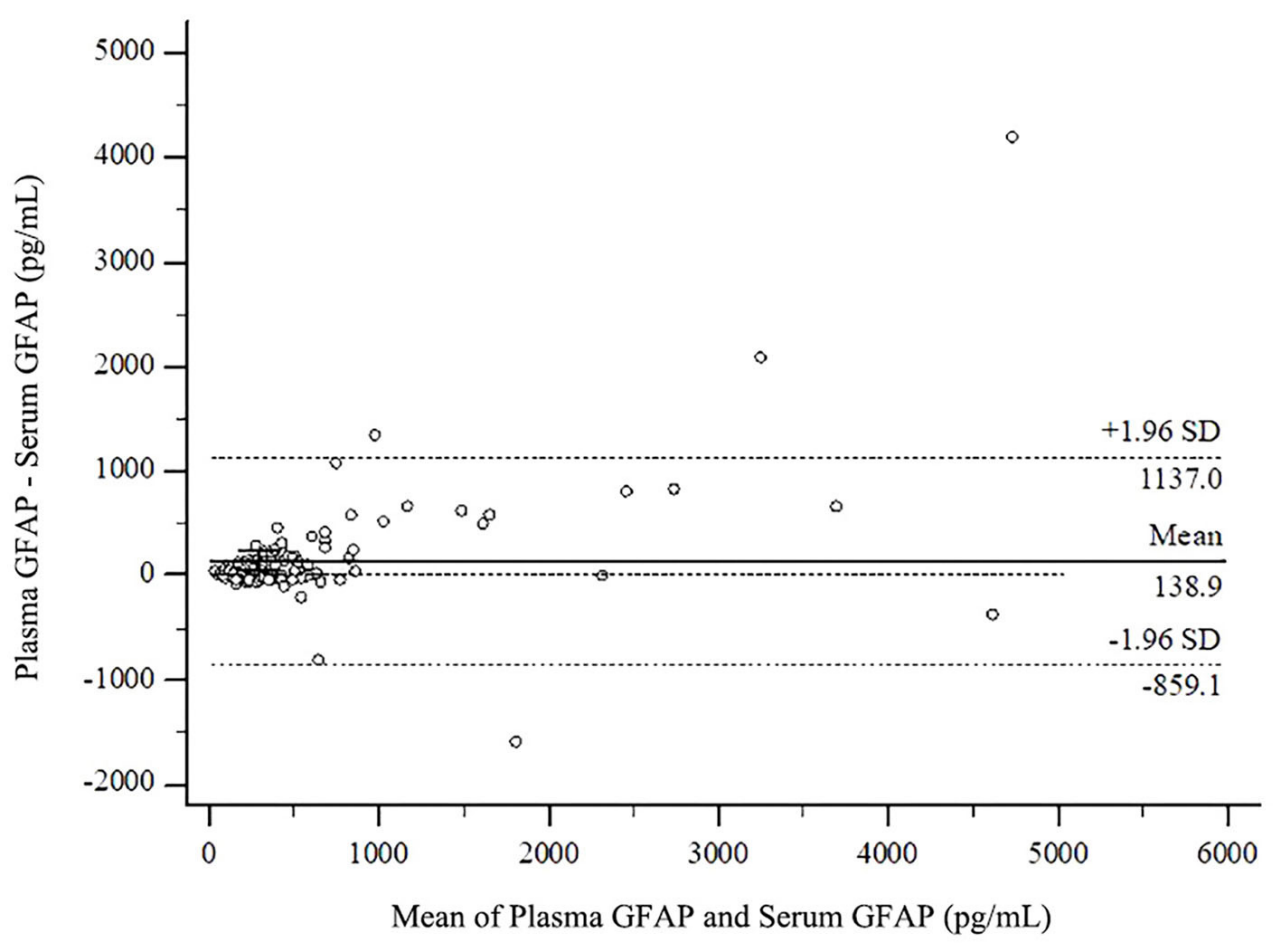
Bland-Altman plot comparing plasma and serum values in the total sample. GFAP, glial fibrillary acidic protein.

A Bland-Altman plot is presented in Figure 1, with the circles illustrating the differences between the plasma minus serum values of GFAP (*N* = 121; *M* = 138.9, 95% CI = 47.3–230.6; *p* < 0.004; lower limit = −859.1, 95% CI = −1,016.2 to −702.0; upper limit = 1,137.0, 95% CI = 979.9–1,294.0; Coefficient of Repeatability = 1,030.5, 95% CI = 915.4–1,179.0). The horizontal dotted lines represent the limits of agreement, in this case defined as the mean difference between plasma and serum GFAP values plus and minus 1.96 times the *SD* of the differences. The line of equality is the dotted horizontal line at 0.0. The mean difference between the two methods is the solid black line (138.9), and the error bars for that line represent the 95% confidence interval for the mean difference. Because the 95% confidence interval does not overlap the line of equality (0.0), there is a systematic difference between plasma and serum GFAP.

**Figure 2:**
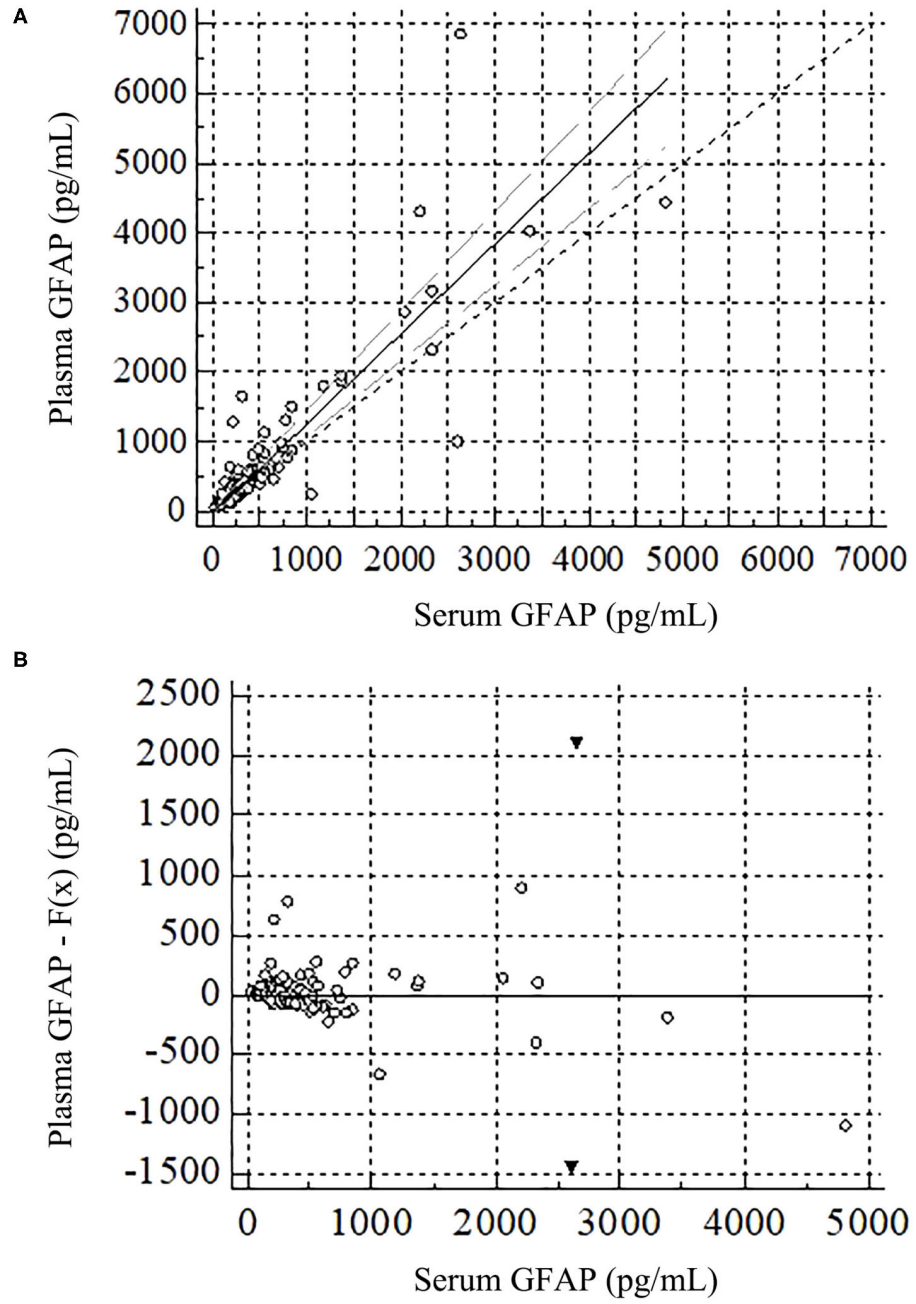
Passing and Bablock regression for plasma and serum GFAP in the total sample. GFAP, glial fibrillary acidic protein. **(A)** Scatter diagram with regression line, confidence intervals, and diagonal line is in the upper figure. **(B)** This residuals plot, in the lower figure, presents the distribution of difference around the fitted regression line, allowing a visual evaluation of the goodness of fit of the linear model. The residuals are the differences between the predicted values and the observed values for the dependent variable. The black triangles represent two extreme values.

A scatter diagram with the regression line and confidence bands is presented in the upper part (part A) of Figure 2. The Passing and Bablock regression equation is *y* = −31.72 + 1.30x; the 95% confidence interval for the intercept value (−31.72) is −50.89 to 7.63 and for the slope (1.30) is 1.10–1.44, revealing a proportional difference between plasma and serum GFAP. The Cusum test, used to evaluate how well a linear model fits the data, revealed no significant deviation from linearity (*p* = 0.49). The residual plot in the lower part (part B) of Figure 2 presents the distribution of differences between predicted values and observed values about the fitted regression line.

In the total sample, 64.5% of patients had higher plasma than serum concentrations. We calculated a difference score between plasma and serum GFAP concentrations by subtracting serum concentration from plasma concentration. The median difference score in the total sample was 28.0 pg/mL. Among difference scores, 17 outliers were identified, defined as GFAP difference scores that were >1.5 times the *IQR*. The characteristics of these participants are presented in Table 3. Twelve had abnormal head CT scans, 4 had normal head CT scans, and 1 did not undergo a head CT scan.

**Table 3:** Characteristics of subjects who were outliers on their GFAP difference scores.

**Age**	**Gender**	**Mechanism of injury**	**Injury to blood sampling (hours)**	**CT result**	**GOS-E outcome**	**Plasma GFAP (pg/mL)**	**Serum GFAP (pg/mL)**	**GFAP difference (plasma – serum)**
80	Male	GLF	1.3	Abnormal	Poor	1,001.8	2,613.8	−1,612.0^*^
89	Female	GLF	2.9	Abnormal	Poor	243.5	1,064.1	−820.6^*^
75	Male	Sport	7.2	Not Done	Poor	4,420.0	4,813.8	−393.8
88	Female	GLF	4.5	Abnormal	Poor	886.4	492.6	393.8
92	Female	GLF	1.4	Abnormal	Poor	630.2	188.6	441.6
89	Female	GLF	4.8	Abnormal	Poor	1,863.0	1,372.2	490.7
81	Female	GLF	3.8	Normal	Poor	1,288.3	788.3	500.0
83	Female	Fall	5.3	Abnormal	Poor	1,941.2	1,382.7	558.5
77	Male	GLF	2.9	Abnormal	Poor	1,792.7	1,191.1	601.5
61	Male	GLF	5.1	Abnormal	Poor	1,490.1	846.6	643.4
89	Female	GLF	3.6	Normal	Poor	4,027.7	3,382.4	645.3
68	Female	Fall	3.3	Abnormal	Poor	2,857.2	2,057.9	799.3^*^
95	Male	GLF	5.9	Abnormal	Poor	3,152.8	2,341.0	811.8^*^
69	Male	GLF	3.1	Normal	Good	1,283.3	218.4	1,064.8^*^
78	Female	GLF	5.4	Normal	Poor	1,651.9	323.2	1,328.7^*^
72	Male	GLF	3.8	Abnormal	Poor	4,296.4	2,212.9	2,083.5^*^
89	Female	GLF	8.3	Abnormal	Poor	6,838.6	2,640.7	4,197.8^*^
						*M* = 2,333.2	*M* = 1,643.0	*M* = 690.3
						*Md* = 1,792.7	*Md* = 1,372.2	*Md* = 601.5

*Outliers were defined as GFAP difference values that were >1.5 times the IQR. Extreme outliers (GFAP difference values >3 times the IQR) are denoted with an asterisk. CT, computed tomography; GFAP, glial fibrillary acidic protein; GLF, ground-level fall; GOS-E, Glasgow Outcome Scale-Extended*.

### Findings by Head CT Group

#### Comparing Normal and Abnormal Head CT Groups

**Figure 3:**
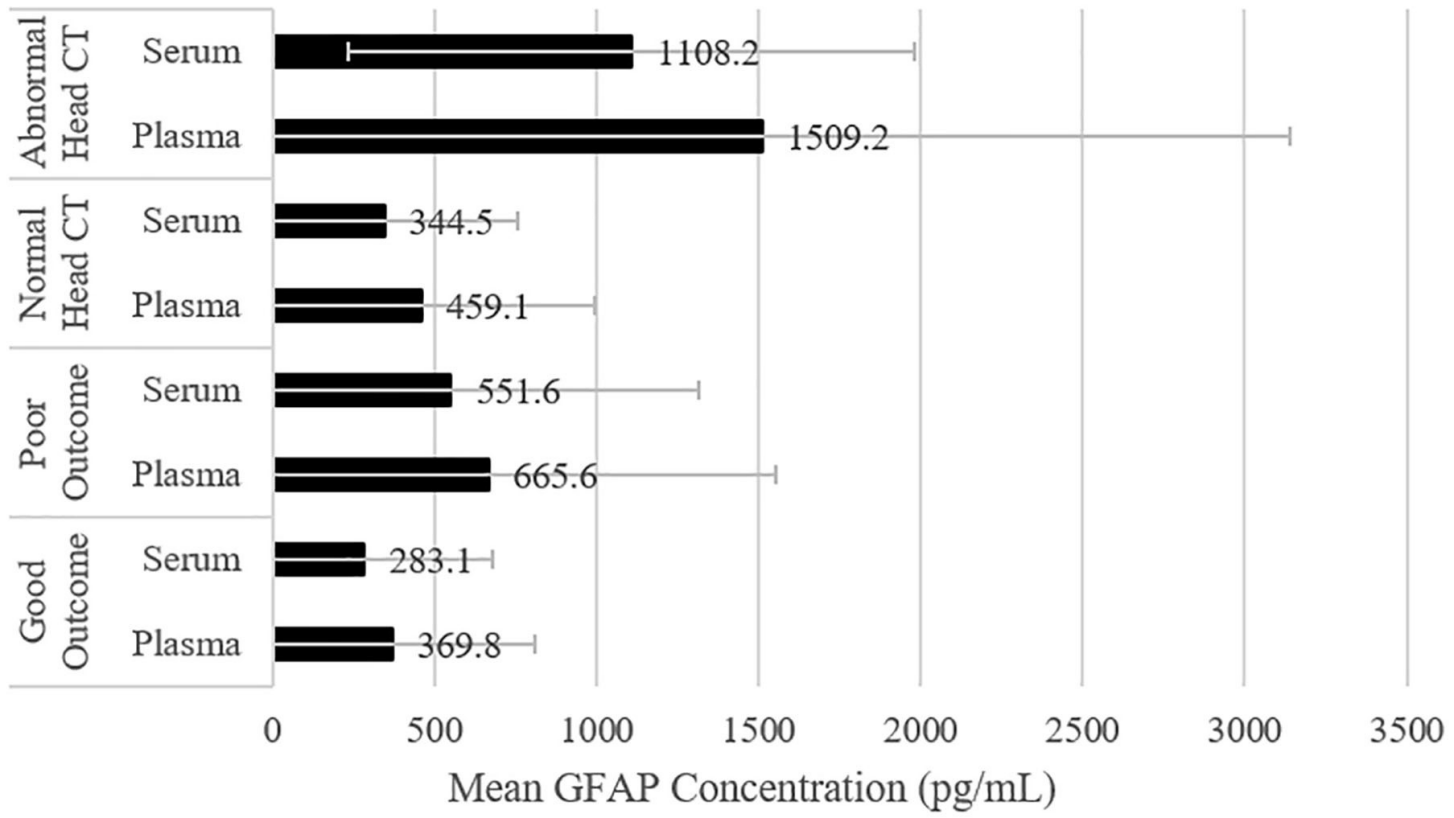
Comparing plasma and serum GFAP levels among subgroups. The mean values are displayed, and the error bars represent one standard deviation. CT, Computed Tomography; GFAP, Glial Fibrillary Acidic Protein.

Serum and plasma GFAP concentrations were first compared *between* the subgroups with normal and abnormal head CT scans (see Table 2 and Figure 3). Both serum and plasma GFAP levels were significantly higher in those with abnormal head CT scans compared to those with normal head CT scans (serum: *U* = 1,253, *p* < 0.001; plasma: *U* = 1,198, *p* < 0.001). ROC curves were computed for serum and plasma GFAP. GFAP levels in serum had slightly greater discriminability than GFAP levels in plasma for detecting intracranial lesions on head CT. The AUC for serum GFAP was 0.814 (*SE* = 0.057, 95% CI = 0.719–0.887, *p* < 0.001) and for plasma GFAP was 0.778 (*SE* = 0.059, 95% CI = 0.679–0.858, *p* < 0.001). The AUC for each fluid was within the 95% CI of the AUC for the other fluid, and a pairwise comparison of the ROC curves revealed no statistically significant difference (*z* = 0.772, *p* = 0.440).

#### Comparing Concentrations Within Subgroups

Serum and plasma GFAP concentrations were then compared *within* head CT subgroups. Stated differently, the concentrations from the two assays were compared for each subgroup. GFAP concentration values were considerably greater in plasma than in serum for the subgroups that had normal (*z* = 4.46, *p* < 0.001, *r* = 0.53, large effect size) and abnormal head CT scans (*z* = 2.09, *p* = 0.036, *r* = 0.45, medium to large effect size). There was no significant difference between GFAP concentration values in plasma and serum for the subgroup that did not undergo head CT (*z* = 0.75, *p* = 0.456, *r* = 0.14, small effect size). Descriptive statistics for GFAP concentration values and difference scores for the CT subgroups are presented in Table 2. GFAP concentration values for these subgroups are presented visually in Figure 3. The correlations (*rho*) between plasma and serum GFAP concentrations within all head CT subgroups were high, but not redundant, and ranged from 0.826 to 0.907. There were similar significant medium positive correlations between both serum and plasma GFAP and age in the subgroup that had normal head CT scans and the subgroup that did not undergo a head CT scan. However, there was no significant correlation between either serum or plasma GFAP and age in the subgroup with abnormal head CT scans (see Table 2).

### Findings by Functional Outcome Group

#### Comparing Good and Poor Functional Outcome Groups

Serum and plasma GFAP concentrations were first compared *between* the subgroups with good and poor outcome (see Table 4 and Figure 3). Both serum and plasma GFAP levels were significantly higher in those with poor compared to good outcome (serum: *U* = 1,625, *p* = 0.002; plasma: *U* = 1,539, *p* = 0.013). ROC curves were computed for serum and plasma GFAP. GFAP levels in serum yielded a slightly higher AUC than in plasma for differentiating between those with good vs. poor outcome; however, neither serum (AUC = 0.690, *SE* = 0.054, 95% CI = 0.584–0.796, *p* = 0.002) nor plasma GFAP (AUC = 0.653, *SE* = 0.060, 95% CI = 0.537–0.770, *p* = 0.013) met AUC cutoffs for acceptable discrimination between good and poor outcome groups. The AUC for each fluid was within the 95% CI of the AUC for the other fluid, and a pairwise comparison of the ROC curves revealed no statistically significant difference (*z* = 1.02, *p* = 0.308).

**Table 4:** Comparison of GFAP concentrations in plasma vs. serum in outcome subgroups.

	**Good outcome**		**Poor outcome**	
	**(*****n*** **= 31)**		**(*****n*** **= 76)**	
	**Plasma**	**Serum**	**Plasma**	**Serum**
**Descriptive statistics (pg/mL)**				
Mean	369.8	283.1	665.6	551.6
Median	253.9	210.6	337.3	300.5
Standard deviation	441.9	397.5	887.0	761.6
Interquartile range	117.1–417.9	119.2–314.5	221.9–621.1	192.4–551.1
Range	63.4–2318.7	48.7–2331.8	52.2–4420.0	32.6–4813.8
**Difference score (plasma – serum)**				
Mean	86.7		113.9	
Median	25.5		38.5	
Standard deviation	205.4		408.7	
Interquartile range	−13.2 to 125.8		−26.0 to 168.2	
Range	−94.4 to 1,064.8		−1,612.0 to 2,083.5	
Percentage with plasma > serum	61.3%		67.1%	
**Group comparisons**				
Wilcoxson signed ranks test (*z*)	2.63 (*p* = 0.009)		3.50 (*p* < 0.001)	
Effect sizes (*r*)	0.47		0.40	
Effect sizes (Cohen's *d*)	0.21		0.14	
**Spearman correlations**				
Age and GFAP level	0.559^**^	0.756^**^	0.435^**^	0.406^**^
Time to blood sampling	0.095	0.196	0.222	0.128
Values from two assays	0.846^**^		0.865^**^	

** *p < 0.01; outcome was defined based on the Glasgow Outcome Scale-Extended (GOS-E), which ranges from 1 to 8, with higher scores indicating better functional outcome. Good outcome was defined as a GOS-E score of 7 (lower good recovery) or 8 (upper good recovery). Poor outcome was defined as a GOS-E score of 6 or lower*.

#### Comparing Concentrations Within Subgroups

Serum and plasma GFAP concentrations were then compared *within* functional outcome subgroups. Stated differently, the concentrations from the two assays were compared for each outcome subgroup. GFAP concentration values were considerably greater in plasma than in serum for subgroups with good (*z* = 2.62, *p* = 0.009, *r* = 0.47, medium to large effect size) and poor outcome (*z* = 3.50, *p* < 0.001, *r* = 0.40, medium to large effect size). Descriptive statistics for GFAP concentration values and difference scores for the outcome subgroups are presented in Table 4, and GFAP concentration values for these subgroups are presented visually in Figure 3. The correlations (*rho*) between serum and plasma GFAP concentrations in both the good (*rho* = 0.846) and poor (*rho* = 0.865) outcome subgroups were high, but not redundant. There were similar, significant, medium positive correlations between both serum and plasma GFAP and age in both outcome subgroups (see Table 4).

## Discussion

### Four Main Findings

This is the first study, to our knowledge, to compare serum and plasma levels of GFAP in a sample of older adults who sustained mTBIs. There were four main findings. First, plasma GFAP levels were significantly higher than serum GFAP levels in the total sample and nearly all subgroups. Second, serum and plasma GFAP levels were highly correlated, but not redundant, in the total sample and all subgroups. Third, GFAP levels measured in both serum and plasma were significantly higher in the subgroup with abnormal head CT scans compared to the subgroup without findings on a head CT scan—with similar ability to discriminate between patients with and without intracranial abnormalities. Finally, GFAP levels in both serum and plasma were significantly higher in the subgroup with poor functional outcome compared to the subgroup with good functional outcome, but both had comparably poor ability to discriminate between those with good and poor functional outcome, warranting larger studies in the future to better determine if these biomarkers combined with others may be of greater value for predicting functional outcome in this population.

### GFAP and Abnormal Head CTs

There is strong evidence that GFAP can be used as a biomarker for identifying people with abnormalities on head CT (1–6, 18, 25–27), and the FDA recently approved GFAP and UCH-L1 for this purpose in the ED setting (24). GFAP outperforms both UCHL-1 (4) and S100B (2) for discriminating between those with normal vs. abnormal head CT scans. In the present study, serum and plasma GFAP had a similar ability to discriminate between those with and without intracranial abnormalities (AUC serum = 0.814; AUC plasma = 0.778). The AUC values in the present study are fairly similar to those reported in other studies (1–6, 18, 25–27, 41) (range = 0.74–0.94) that have examined how effectively GFAP discriminates between patients with and without intracranial abnormalities following TBI (in samples of patients with predominantly mild injuries).

### GFAP and Age

There is evidence that GFAP is elevated differentially in older adults following TBI (31, 32). In the present study, there were positive correlations between GFAP and age, in both serum and plasma, in the total sample and in nearly all subgroups. Thus, GFAP levels are greater with greater age. In most subgroups, the correlations between GFAP and age were modestly larger in serum than in plasma. One possible explanation is that fibrinogen and other clotting factors in plasma influence the correlation between GFAP and age (42). Interestingly, there was no correlation between GFAP levels and age in the subgroup with abnormal head CT scans. Past researchers examining GFAP levels following TBI (93.3% mild in severity) found that among those with intracranial abnormalities (i.e., CT positive), GFAP concentrations did not significantly differ between younger (<65 years old) and older (≥65 years old) adults (32). However, among the total sample and those with normal head CT scans, older adults had significantly higher median GFAP levels than younger adults (32). Therefore, it is possible that neurotrauma resulting in macroscopic intracranial abnormalities reduces or even obliterates the association between GFAP levels and age, although this is speculative and cannot be evaluated in the present study.

### GFAP and Functional Outcome

There is modest evidence that GFAP is associated with functional outcomes following TBI (7–10). Prior studies have reported adequate ability (i.e., AUC ≥ 0.70) of GFAP for discriminating between those with favorable and unfavorable outcomes following TBI (4, 8, 27, 43). However, inclusion of patients exclusively with moderate-to-severe TBI (8) and less stringent definitions of good vs. poor outcome (e.g., GOS-E >4 = good outcome) (4, 27, 43) may have contributed to those findings. In the present study, we found that those with worse outcome had greater levels of GFAP in both serum and plasma. However, our AUC values were not significant, illustrating that GFAP could not adequately discriminate between these groups. Our study, however, had a very limited outcome assessment—we examined global outcome at 1-week post-injury. It remains unclear whether GFAP, in isolation, is clinically useful for predicting prognosis or functional outcome following mTBI. In a study of adults with mostly mild injuries, although GFAP was associated with functional outcome, GFAP did not predict unfavorable outcome in a multivariate regression model in which age, GCS score, and Marshall score were significant predictors (10). Biomarkers with differing cellular origins and temporal dynamics likely contribute differently to predicting recovery, and panels of several different biomarkers have been shown to improve outcome prediction following *severe* TBI compared to single biomarkers alone (44).

### Plasma vs. Serum GFAP

The findings in this study for serum and plasma levels of GFAP were similar, but not identical. The levels were highly correlated, but not redundant, with most correlations ranging from 0.83 to 0.91 (Table 2). The reason for higher GFAP levels in plasma compared to serum is unclear. One possible explanation is that GFAP may become trapped in the fibrin-platelet matrix during clotting, which could account for lower levels of GFAP in serum compared to plasma (45, 46). GFAP levels in plasma and serum did not significantly differ for the subgroup that did not undergo head CT. It can be speculated that the trauma-induced coagulopathy was more severe in the patients who underwent CT scanning. Fibrin-platelet trapping is less likely in very mildly injured individuals, which could account for the lack of difference between serum and plasma GFAP levels in those who did not undergo head CT. In addition, the levels were measured using different assays, which could result in systematic bias of measurement, because the assays were not standardized to a common calibrator or a certified reference material. Different assays might also have different sensitivity to interferants that might be present in samples. The plasma samples in the present study were frozen and analyzed 1.5 years after the serum samples were analyzed, but the possible effect of this greater time in the freezer is thought to be negligible—especially because there was only one freeze-thaw cycle. There may also be matrix-related differences between plasma and serum of relevance to GFAP as a biomarker. Researchers examining a sample of adults with intracranial abnormalities following TBI (90% mild in severity) reported higher median GFAP levels in plasma than serum over 72 h after blood being drawn; however, the researchers did not statistically compare GFAP levels between fluids and the plasma and serum concentrations were nearly perfectly correlated (*r* = 0.994) (47).

### Limitations

We ran the biomarker analyses in singlicates. This method does not allow us to account for variation within the assays. However, for both plasma and serum GFAP, two quality control samples were run in duplicates in the beginning and end of each run. These quality control samples revealed low analytical variation for both assays and gave us reasonable confidence that running the samples in singlicates was appropriate.

Our study had small sample sizes in the abnormal head CT group and the group who did not undergo a head CT. When examining the two CT subgroups comparing plasma and serum levels, we had small sample sizes which reduces power. For the no head CT group (*n* = 29, *d* = 0.001), we had very low power (just 0.05 based on a *post-hoc* power analysis), but the low power is due to an extremely small effect size. As such, it is reasonable to accept the null hypothesis for this particular analysis. Considering the small effect, it is not likely that serum and plasma differ in this group. For the abnormal CT group (*n* = 22, *d* = 0.31), we observed a significant group difference, indicating that low power did not interfere with finding a significant effect for this analysis, despite a small sample size.

Given our small sample size of those with abnormal head CT scans, we were not able to further investigate why there was no significant correlation between GFAP and age in this subgroup. With a substantially larger sample of people with abnormal head CT scans, it would be possible to do a more careful analysis of the association between the specific types of abnormalities and lesion loads in relation to GFAP and age.

### Conclusions

In conclusion, in a cohort of older adults following mTBI, GFAP levels were highly correlated in serum and plasma, and GFAP had similar ability to discriminate between individuals with and without intracranial abnormalities. Both plasma and serum GFAP levels had inadequate ability to discriminate between individuals with good and poor global functional outcome at 1-week following injury. Taken together, these findings suggest that the clinical impact of testing GFAP levels in plasma vs. serum is small. However, it is possible that the differences in fluid concentrations may have clinical significance in differential diagnosis between some patient groups in different clinical settings and are worthy of additional study. Plasma and serum GFAP were correlated with age in the total sample and all subgroups except the group with abnormal head CT scans. Additional research is needed to determine if neurotrauma resulting in intracranial lesions reduces or eliminates the correlation between GFAP and age.

## Data Availability Statement

The raw data supporting the conclusions of this article will be made available by the authors, without undue reservation.

## Ethics Statement

The studies involving human participants were reviewed and approved by Ethics Committee of Pirkanmaa Hospital District, Tampere, Finland (ethical code: R15045). The patients/participants provided their written informed consent to participate in this study.

## Author Contributions

NH assisted with the literature review, the statistical analyses, writing of the manuscript, editing drafts of the manuscript, and he approved the final version for submission. TL was the principal investigator on the parent study, of which we used the data for secondary analyses. He contributed to the patient enrollment, literature search, reviewed drafts of the manuscript, and approved the final version for submission. JK assisted with the literature review and statistical analyses, edited drafts of the manuscript, and approved the final version for submission. KBe reviewed and coded the head CT scans, and approved the final version for submission. KBl and HZ co-supervised the laboratory in which the biomarker analyses were conducted and secured funding for the analyses, edited drafts of the manuscript, and approved the final version for submission. NA and JS assisted with running the biomarker analyses, edited the manuscript, and approved the final version for submission. JP and JG assisted with the literature review, edited drafts of the manuscript, and approved the final version for submission. GI conceptualized the study, conceptualized and ran the statistical analyses, wrote sections of the manuscript, and approved the final version for submission. All authors contributed to the article and approved the submitted version.

## Conflict of Interest

KBl has served as a consultant, at advisory boards, or at data monitoring committees for Abcam, Axon, Biogen, Julius Clinical, Lilly, MagQu, Novartis, Roche Diagnostics, and Siemens Healthineers, and is a co-founder of Brain Biomarker Solutions in Gothenburg AB (BBS), which is a part of the GU Ventures Incubator Program. HZ has served at scientific advisory boards for Denali, Roche Diagnostics, Wave, Samumed, Siemens Healthineers, Pinteon Therapeutics and CogRx, has given lectures in symposia sponsored by Fujirebio, Alzecure and Biogen, and is a co-founder of Brain Biomarker Solutions in Gothenburg AB (BBS), which is a part of the GU Ventures Incubator Program. GI serves as a scientific advisor for BioDirection, Inc., Sway Operations, LLC, and Highmark, Inc. He has a clinical and consulting practice in forensic neuropsychology, including expert testimony, involving individuals who have sustained mild TBIs. He acknowledges unrestricted philanthropic support from ImPACT Applications, Inc., the Mooney-Reed Charitable Foundation, and the Spaulding Research Institute. The remaining authors declare that the research was conducted in the absence of any commercial or financial relationships that could be construed as a potential conflict of interest.
